# Quantum-inspired modeling of social impact in complex networks with artificial intelligent agents

**DOI:** 10.1038/s41598-025-22508-y

**Published:** 2025-10-08

**Authors:** A. P. Alodjants, D. V. Tsarev, P. V. Zakharenko, A. Yu. Khrennikov, A. V. Boukhanovsky

**Affiliations:** 1 ITMO University, 197101 St. Petersburg, Russia; 2 International Center for Mathematical Modeling in Physics, Engineering, Economics, and Cognitive Science Linnaeus University, S-35195 Vaxjo-Kalmar, Sweden

**Keywords:** Complex networks, Complex networks, Mathematics and computing, Physics, Psychology, Psychology

## Abstract

We propose a quantum-inspired framework for modeling open distributed intelligence systems (DISs) comprising natural intelligence agents (NIAs) and artificial intelligence agents (AIAs) that interact with each other. Each NIA – AIA pair represents a user and their digital assistant – an avatar implemented as an agent based on a large language model (LLM). The AIAs are interconnected through a complex, scale-free network and communicate with users and one another in real time. We focus on the social impact and evolution of users’ emotional states, which we model as simple, two-level cognitive systems shaped by interactions with AIAs and external information sources. Within this framework, the AIAs adiabatically follow the NIAs, mediating emotional influence by disseminating information and propagating user emotions throughout the system. Building on Mehrabian’s Pleasure–Arousal–Dominance (PAD) model and Wundt’s three-dimensional theory of emotions, we put forward a quantum-like representation of affective states on an emotional sphere. We demonstrate that the arousal component is governed by the interplay between external informational inputs and individual personality traits. This leads to the emergence of limiting cycles in emotional dynamics. Assuming weak AIA – AIA coupling, we identify two distinct regimes of affective behavior. In the first regime, coherent NIA – AIA interaction supports emotional heterogeneity and individual differentiation across the network. In the second regime, shared exposure to external information drives synchronized emotional responses, resulting in a macroscopic affective field that captures collective emotional dynamics. Furthermore, we demonstrate that the network’s structural properties, particularly node degree correlations, play a role analogous to quantum correlations in ensembles of two-level physical systems; a quantum-like superradiant state corresponds to the network-induced collective emotional activation of NIAs within a DIS. These findings advance our understanding of affective dynamics and emergent social phenomena in hybrid human–AI ecosystems.

## Introduction

### Background and motivation

An individual’s emotional state, or mood, is an indispensable part of their daily life. Emotions significantly affect people’s behavior, interpersonal relationships, and decision-making (DM)^1^. The development of IT and the Internet pushed the interest in studying human emotions, their description, modeling, and dissemination, making them a subject of intense debates, e.g.^2,3^, for two main reasons.

First, in social networks, individuals closely interact, spending a lot of time in various online communities, solving business, educational, informational, leisure, and other tasks. The individuals’ communication in networks promotes their mutual influence with emotions, which can increase during the distribution. As a result, a certain emotional background is created throughout the community and can be easily traced by the tonality of the information distributed, for example, posted on a community wall. These issues are important for modern societies, since in some cases, the intensification of emotional states is undesirable and even dangerous if it contributes to the spread of hatred and envy.

Second, the problem of identifying emotional states in network communities has become relevant in the context of artificial intelligence (AI). Current AI agents (AIAs) no longer operate solely by following predefined rules or relying on a limited set of functions; a comprehensive overview of the evolution of cognitive architectures for agents of both artificial and natural intelligence over the past four decades is given in^4^, and cf.^5^. Temporary social networks establish so-called Distributed Intelligence Systems (DISs) as a new paradigm for modern society organization^6^. The rapid development of socially oriented networks in the AI environment has made them an essential part of information exchange. DISs can be recognized as a mixture of natural (human) and AIAs, cf.^7^. AIA can take the form of digital assistants (avatars, chatbots), but it can also encompass more complex entities that relate to so-called AI foundation models^8^. Modern large language model (LLM) -based bots, built on transformer architectures and trained on large-scale datasets, are capable of interpreting meaning, understanding dialogue context, recognizing intent, and detecting the emotional states of other agents. These systems can sustain long-term conversations, generate original content, summarize interactions, and draw inferences, see, for example,^9–11^. Thanks to their remarkable abilities in language creation, knowledge utilization, and complex reasoning, LLMs are becoming an increasingly indispensable part of multi-agent system exploration^12–15^. Obviously, the interaction of LLM-based agents with humans within the DIS can also reveal the properties of emotional intelligence. Note that LLMs can also be used to identify and manage emotions within the networks^16–18^.

Although finding optimal topologies of DIS involving LLM-agents and humans is of particular interest (cf.^15^), this work analyzes complex graphs as a prototype of the networks usually inherent to NIA communities, cf.^19^. Complex networks that pose hubs (maximal connectivity nodes) resemble the main features of social networks, cf.^20,21^; an important feature is the synchronization abilities^22^ that allows complex spreading effects and social impact^23–25^. Notably, AIAs based on the current capabilities of AI foundation models can carry emotional states and contribute to the formation of certain moods in the DIS^18^. In this regard, the DM problem in such DISs should take into account the emotional intelligence that modern AIAs can demonstrate^16,17^. Thus, the problem of the creation, formation, and spreading of collective emotions and moods in DISs established by complex networks represents a remarkable and challenging task.

At the same time, it is important to recognize the challenges faced by modern LLM-based AI agents (AIA). These models often have an excessive tendency to align with the user’s perspective after just a few interactions, even when that perspective is factually incorrect or logically flawed^26,27^. LLM agents such as GPT-4 are trained on vast amounts of data, enabling them to generate contextually appropriate responses that align with user expectations. While this behavior can be beneficial in scenarios where maintaining a positive and coherent tone is desirable, it also highlights a fundamental limitation. LLMs lack critical reasoning based on a true understanding of the world, and they cannot independently verify factual information. As a result, they tend to ’adiabatically’ follow the prevailing discourse and tone of interactions with NIA. This characteristic of LLM-based AIAs is precisely what we use in this study.

In this paper, we aim to address this problem by continuing our previous research^28–31^. In particular, in^28^ we suggested a laser-like (open, driven-dissipative) model for multi-agent DM in complex networks. Our approach, established in^28,30,31^ is based on macro-level modeling (the so-called system-based simulation^32^,) of the open DIS, where equations are used to describe the dynamics of the information field, agent system activation (social field parameter), and agents population differences–variables that can be naturally associated with the system as a whole. At the same time, we introduce a set of simple behavioral rules for NIA, which, for the sake of simplicity, are endowed with a two-level cognitive structure. The quantum-like approach enables the accounting for spontaneous processes involving DM agents, which can, in a sense, be associated with these agents’ “free will”, cf.^33^. Ultimately, these processes are responsible for the irrational decisions made by NIAs (i.e., humans) in real life, see e.g.^34,35^. We described NIAs as two-level quantum-like systems possessing binary decisions under environment-supported uncertainty, cf.^36,37^. Based on the elementary DM processes of agents in the network, we obtained conditions for the threshold of information dissemination in the network. Our results are in good agreement with other models, which predict epidemic spreading and cascades^38^, but our model also takes into account particular features of individual agents in DM under uncertainty at the microscopic level. Then, in^30^, we applied our results for modeling the DISs representing more complicated systems.

This study presents a model of an open DIS consisting of users and their avatars connected by an AIA–AIA graph. We first introduced this type of DIS in^29^. Using classical agent-based modeling (ABM), we investigated the potential for AI agents (avatars and bots) to adapt to their human users. The model was validated empirically in student groups during the COVID pandemic. As students had access to external resources (e.g., literature) and could communicate with friends outside the system, the DIS functioned as an open system. The primary objective of the research conducted in^29^ was to support students in adapting to online learning. However, the experimental results showed that avatars (bots) did not always remain synchronized with their users in the long term. Instances of decoupling were observed where the avatars began to diverge from the users’ behavior and exhibited autonomous self-organization.

In^30^, we proposed a relatively simple quantum-like two-level model that allows the DIS theoretical description. We showed that AIA suggestions made for users reflect the average “collective mind” inherent to AIA. Thus, AI agents are self-organized within the complex network through the information exchange and AIA – AIA interaction facilities. Crucially, our approach is based on modeling strongly non-equilibrium (laser-like open systems), which contrasts with the traditional (equilibrium) statistical physics methods usually employed to simulate complex networks under thermodynamic equilibrium, cf.^39^.

It is interesting to note that the non-trivial influence of topological properties of complex networks on the collective behavior of DM agents in social networks has recently been confirmed by studies based on ABM, see^40^. The system investigated in^40^ serves as a representative example of an open DIS examined in our works^28,30^ by means of a social field approach. In particular, in^40^, the authors researched the negative effects arising from the influence of external social media. In this study, the authors examine a range of effects, including social polarization, attention inequality, and the amplification of extreme opinions in social networks. These effects are modeled through ABM incorporating LLMs. Specifically, agents were constructed based on profiles from the American National Election Studies, augmented with LLMs to generate biographical narratives and simulate behavioral responses. The^40^ demonstrates that ideological homophily, attention inequality, and the amplification of extreme voices can emerge even in the absence of recommendation algorithms – that is, in a minimalist environment where agents merely post, repost, and follow one another. Larooij and Törnberg attribute these phenomena, at a fundamental analytical level, to the coupled dynamics of content engagement and the evolving topology of social networks.

The purpose of this work is to demonstrate that AIAs enable the dissemination of “collective mood” within the complex network affecting users’ emotional states, cf.^16,17^. In this sense, the results of our previous work^31^ related to the formation of collective emotions will be revised at a new level, accounting for new paradigms with AI foundation models^8^. Namely, in^31^, we proposed a description of the model of individuals’ emotions for a social system at thermal equilibrium with the environment. We showed that the formation of collective emotions can be recognized as a superradiant state formation in quantum physics^41^. It will be demonstrated that, in this case, AIA, adiabatically following their users, act solely as carriers of emotions within the DIS, cf.^42,43^. However, at its core, the social system under consideration is open, driven-dissipative. As a result, the vital problems of interaction of DM agents with the environment and the influence of the specific topology of network structures, in which these agents are placed, remain open.

In this work, first, we propose a quantum-like approach for describing emotional states of individuals in 3D in the presence of their interaction with the environment. We emphasize the symmetry properties that this approach provides. Second, we consider a more realistic situation in which individuals are placed within the DIS and interact with each other through coupling with their LLM-based agents. The main task here is to clarify the influence of the network environment on the emotional states of individuals.

The article is arranged as follows. In the Background and Motivation and Related works subsections, we establish the motivation and significance of our study for social sciences; we also review relevant literature for the problems under discussion. In the Model and Methods section, we suggested the quantum-like model of emotional states and established the methods and approaches we use to obtain a set of mean-field equations examined in this work. In particular, in Quantum-like 3D model of feelings and emotions subsection, we demonstrate how a 3D model of emotions and Wundt’s model of feelings can be mapped onto the Bloch sphere typically used for the description of two-level (or 1/2 spin) systems in two-dimensional Hilbert space. To be more specific, we restrict ourselves to the so-called pure quantum-like state description of basic emotions. In the Open DIS model subsection, we establish our model of DIS. In particular, in DIS network we specify the DIS complex network that presumes AIA – AIA coupling; we approach the network by a power law degree distribution. In Quantum-like model of DIS, a quantum-like description of DIS is given; we represent the Hamiltonian of DIS and specify the main parameters we use for NIAs and AISs in DIS. In the Mean-field equations subsection, we discuss and analyze the main equations within the mean-field approach. In the Results section, we establish the main results of our work related to quantum-like simulation of emotion dissemination within open DIS. The Network-free individuals emotional states, $J=0$ subsection establishes our results on NIA’s emotional state recognition by using our approach. In this part of the work, we exclude the influence of the network to find out which processes affect the change in the state of emotions and moods of individuals. The Collective emotions in the presence of weak AIA – NIA coupling subsection represents the essence of our results. We demonstrate how collective emotions and moods can be established and disseminated within the DIS, which represents an open (driven-dissipative) system. We obtain relevant conditions for the enhancement of collective moods in our work. The role of NIA – AIA pairs synchronization is elucidated. Especially, we point out a steady-state superradiant laser approach that represents a useful tool for our purposes here. In Conclusions section, we summarize the results obtained.

### Related works

#### Agent-based modeling approach

To gain a deeper understanding of the issue of emotional intelligence modeling, it is helpful to start with the ABM approach and review the emotion models currently being investigated in cognitive science, see, for example^44^,. It is important to note that early proposed models are mostly two-dimensional. In particular, we emphasize Russell’s circumplex model, which is quite widespread in current studies^45^. For this model, emotions are represented on a circle providing arousal-valence variables. The Plutchik model allows for description in addition to the intensity of emotions through various color domains arranged within the circle^46^.

With the development of the Internet, the recognition of individuals’ emotional states through various signatures of their behavior in online communities has become an important area of theoretical^31^ and applied research^43,47^. In the context of emotion modeling, a considerable body of research focuses on micro-level modeling of network social systems through ABM, where the overall system behavior emerges from the interactions of individual agents exhibiting emotional responses. These models are discrete in nature, analogous to cellular automata, with agent behavior and interactions governed by specific rules or parameterized models^42,48–50^. To model the dynamics of complex agent-based systems, the authors of^3,51–53^employed the ABM approach, incorporating time-discrete changes in agent characteristics such as knowledge and emotions, as well as activity profiles (e.g., number of actions within a given time slot, characteristic time delays, and circadian cycles). An advantage of this approach lies in its ability to derive macroscopic system properties–such as self-organization–from the specific attributes assigned to individual agents. In particular, to describe emotions in networks involving bots, several studies utilized Russell’s two-dimensional model of arousal and valence, cf.^42,43,49^. Based on the ABM approach, these works demonstrated the influence of emotionally-oriented bots on network agents, allowing for the investigation of collective emotional dynamics in online chat environments. The findings show that emotionally-oriented bots can significantly alter the global emotional state of agents in the network, triggering cascade-like shifts in mood.

Due to the variety of network-oriented social communities, we can find further development and extension of Russell’s circumplex model, driven by the need to provide a more complete picture of individuals’ emotional moods^54^. At the same time, higher-dimensional models of emotions are becoming more and more popular^55^. For the approaches considered in this paper, 3D models are of particular importance for the classification of emotional states. Among such 3D models is Mehrabian’s PAD (Pleasure-Arousal-Dominance) model as the most important^56^. In this model, the dominance dimension is used to decide whether the subject feels in control of the state or not. The pleasure dimension is similar to valence in Russell’s model. It is interesting to note that Wundt once pointed out the need to use 3D models in psychology^57^. In particular, according to Wundt’s approach, our feelings can be minimally represented within a three-dimensional model by using the basic states of pleasant-unpleasant, strain-relaxation, and excitement-calm. In this case, specific feelings (emotions) can be expressed through combinations of these states. Fortunately, both Wundt’s and Mehrabian’s approaches can be mathematically described very convincingly within the quantum probability theory formalism. These approaches can be visualized clearly using the Bloch sphere to describe the cognitive state of both individuals and social groups, cf.^58,59^. Furthermore, we show that our proposed description of emotional states makes it possible to determine the intensity of emotions as well as their change over time.

#### System-based modeling approach

To understand the fundamental role of emotions in the description of current social processes based on quantum approaches to probability theory, it is useful to address the field, a key concept in physics^60,61^, and also used in the social sciences^62^. This approach relies on macroscopic modeling of complex systems, accounting for the specific characteristics of the evolution of physical, informational, or social fields^32^,. In physics, of primary importance is the key discovery of the laser as a coherent source of an electromagnetic field, which leads to the study of matter-field interaction^63^. At the same time, the laser field plays an indispensable role in optical communication as a carrier of information^64^. The concept of field was first proposed in cognitive science over a hundred years ago^65,66^. In the works, inspired by physical field models, the concept of personality is associated with the concept of the field as the unity of personality and its environment. Lewin claims that mental energy is transferred from a person to their surroundings, which acquire certain characteristics as a result. The concept of field in sociology was developed by Bourdieu^67,68^. According to his theory, the social field is not created by the number of elements, but by the totality of relations and connections between them, which cannot be reduced to the intentions of individual agents, nor their interaction. In terms of physics, here we deal with the phenomenon of interference, which can manifest itself in social fields^67^. In modern social sciences, the field theory studies the different ways individuals create social fields and how these fields are influenced by them^69^.

Based on the laser analogy, in^63,70^, the authors pointed out the interdisciplinary nature of coherent effects that may be important for a system of interacting individuals. The Internet and especially social networks have created fundamentally new prerequisites for the development of the social field and social impact concepts^71^. It is shown that current social network communities enable the formation of coherent information fields, as it occurs in physical lasers, cf.^72,73^. In^28^, we showed that a scale-free network of DM agents with binary choices provides a useful environment for social reinforcement under certain conditions. The term “social laser” in this case fully reflects the social processes of strong enhancement and impact, which can appear in the presence of a social network environment. The idea of a social laser is quite simple and close to a physical laser paradigm. Individuals who are already linked in a network receive a lot of information from the environment within a short period of time, which in physics is called pumping. This creates an extremely excited state of society, which then becomes capable of releasing a huge amount of social energy. Social networks with scale-free properties can significantly reinforce information during this process, which contributes to social enhancement of action. Importantly, the observed results do not refer to the emotions of individuals. The explanation for this phenomenon is as follows.

If the process of information pumping is fast enough, then the emotions of individuals change and follow the information field. Mathematically, this means that some social system characteristics (its polarization) adiabatically follow the field^28,63^. However, the opposite often happens in practice: the emotions of individuals determine their further actions, and the information field follows social polarization. This is exactly what occurs in superradiance in quantum physics^41^. In this case, the quantum correlations between dipoles (two-level) systems represent a significant ingredient for collective polarization state formation. The *N* two-level systems radiate an optical field into the cavity at a rate proportional to collective polarization, i.e., the optical field follows the polarization of two-level systems (TLSs). The intensity of radiation is enhanced and proportional to $N^2$,^41^. Noteworthy, current experiments on superradiance demonstrate that a physically small number of photons provides correlation (synchronization) of a macroscopically larger number of dipoles representing TLS excitations^74^. Thus, the collective polarization of quantum two-level systems plays a key role in the superradiance effect. To clarify this issue, it is instructive to refer to the polarization definition in optics.

Consider the properties of a photonic field, which can be analogous to certain social processes; we can assign amplitude and phase field variables. The amplitude of the field characterizes the intensity of the radiation, while the phase of the field determines its coherent properties; they are sufficient to fully describe both physical and social lasers^28,63^.

However, the vector nature of the photonic field requires an additional degree of freedom. Polarization of radiation in physics represents a necessary ingredient to understand the vector nature of both classical and quantized electromagnetic fields. The concept of polarization can also be extended to the fields of massive particles; for example, the polarization of an electron appears due to its spin properties^61^. Therefore, in physics, polarization is an additional degree of freedom describing a field^60^.

For our purposes, it is important that in quantum theory, polarization can also be prescribed to massive particles like electrons, atoms, etc. In this case, polarization can occur as a 1/2 spin particle or two-level system with specific features that satisfy the *SU*(2) algebra symmetries, cf.^75^. Geometrically, the polarization state can be easily established in three dimensions employing a representative point on the Poincaré sphere in optics^60^ or the Bloch sphere for 1/2 spin systems^76^.

Thus, in this work, we specify the emotions of individuals by analogy with the description of the polarization of the physical field. In this case, emotions constitute an intrinsic property of the social field and serve as additional “degrees of freedom” in the main features of social processes. As a result, the quantum-inspired superradiant state analogue represents some specific emotionally activated state in humans.

## Model and methods

### Quantum-like 3D model of feelings and emotions

#### The model of feelings and emotions

At first, let us consider the “pure” emotional states’ specification, which presumes the absence of any interaction of individuals with the environment. Descriptions of an individual’s emotional states, in most cases, are based on opposing feelings. The pairs of opposite primary emotions (acceptance – disgust, joy – sadness, anger – fear, anticipation – surprise) appear in the complex models, e.g.^44,77^. Physically, these emotions can be formulated in terms of a quantum-like two-level system or a system with “spin up” and “spin down” variables. We recognize NIAs as so-called social atoms, which pose two basic mental states $|0\rangle$ and $|1\rangle$, cf.^36^. In this case, for a description of emotional states, it is useful to refer to the quantum probability theory approach, which specifies a pure qubit-like affective state in the form

1 $$\begin{aligned} |\Psi \rangle = \cos (\theta /2) |0\rangle + e^{i\varphi } \sin (\theta /2)|1\rangle , \end{aligned}$$

where $\theta$ and $\varphi$ are two phase angles that we specify later; $0\le \theta \le \pi$; $0\le \varphi <2\pi$. In (1) $|0\rangle$ and $|1\rangle$ are two mutually orthogonal states in a *two-dimensional* Hilbert space that obey the condition

2 $$\begin{aligned} \left\langle 1|1\right\rangle = \left\langle 0 |0\right\rangle = 1, \quad \left\langle 1 |0\right\rangle = 0. \end{aligned}$$

In quantum information, $|0\rangle$ and $|1\rangle$ specify a so-called computational or measurement basis^76^. Notably, we can also identify other pairs of states as

3a $$\begin{aligned} |\pm \rangle&= \frac{1}{\sqrt{2}}(|0\rangle \pm |1\rangle ); \end{aligned}$$

3b $$\begin{aligned} |\pm i\rangle&= \frac{1}{\sqrt{2}}(|0\rangle \pm i|1\rangle ). \end{aligned}$$

In the framework of emotional state specification, we can recognize emotional basic states (3) as

4a $$\begin{aligned}&|1\rangle \equiv |\textit{Aroused}\rangle , \quad |0\rangle \equiv |\textit{Unaroused}\rangle ; \end{aligned}$$

4b $$\begin{aligned}&\quad |+\rangle \equiv |\textit{Pleased}\rangle , \quad |-\rangle \equiv |\textit{Annoyed}\rangle ; \end{aligned}$$

4c $$\begin{aligned}&\quad |+i\rangle \equiv |\textit{Dominant}\rangle , \quad |-i\rangle \equiv |\textit{Submissive}\rangle . \end{aligned}$$

Thus, in Eqs. (4) we characterize quantum-like emotional state $|\Psi \rangle$inherent to individuals as a generic quantum-like TLS that exploits Mehrabian’s basic emotional states in 3D, cf.^55,56^. Noteworthy, our approach also admits coding for three-dimensional Wundt’s model of feelings (cf.^57^) as

5a $$\begin{aligned}&|1\rangle \equiv |\textit{Excitement} \rangle , \quad |0\rangle \equiv |\textit{Calm}\rangle ; \end{aligned}$$

5b $$\begin{aligned}&\quad |+\rangle \equiv |\textit{Pleasantness}\rangle , \quad |-\rangle \equiv |\textit{Unpleasantness}\rangle ; \end{aligned}$$

5c $$\begin{aligned}&\quad |+i\rangle \equiv |\textit{Strain}\rangle , \quad |-i\rangle \equiv |\textit{Relaxation}\rangle . \end{aligned}$$

**Fig. 1:**
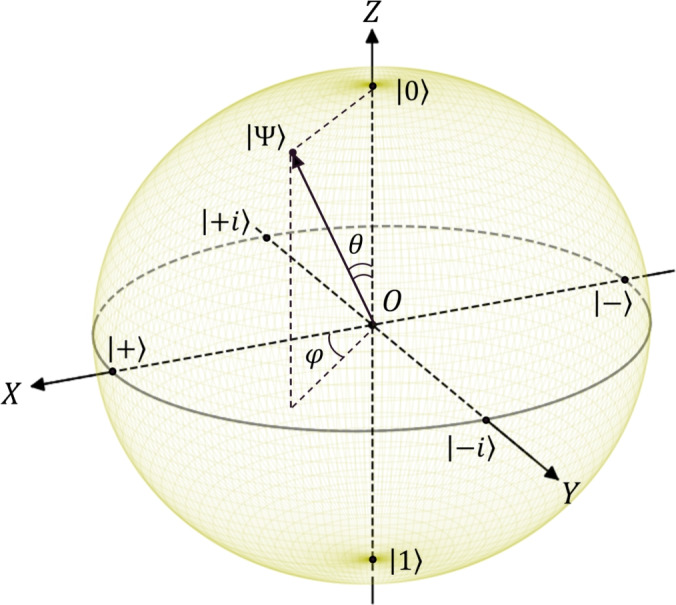
Representation of emotional state in Bloch sphere as a quantum-like qubit state. The details are explained in the text.

Fig. 1 demonstrates * affective sphere* in the form of the Bloch sphere for specification of 3D emotions in terms of the pure state $|\Psi \rangle$, see (1), (4). To describe the change in the emotional state defined by vector $|\Psi \rangle$, it is necessary to define operators acting in the Hilbert space with states (4). Thus, we specify operators

6a $$\begin{aligned} \hat{\sigma }^{+}&= |1\rangle \langle 0|; \end{aligned}$$

6b $$\begin{aligned} \hat{\sigma }^-&= |0\rangle \langle 1| =(\hat{\sigma }^{+})^{\dagger }; \end{aligned}$$

6c $$\begin{aligned} \hat{\sigma }^{z}&= |1\rangle \langle 1| - |0\rangle \langle 0| \equiv \hat{n}^{(1)} - \hat{n}^{(0)}; \end{aligned}$$

6d $$\begin{aligned} \hat{\sigma }_{0}&= |1\rangle \langle 1| + |0\rangle \langle 0| \equiv \hat{n}^{(1)} + \hat{n}^{(0)}. \end{aligned}$$

It should be noted that the arousal states defined in (4a) allow the operator $\hat{\sigma }^{+}$ in (6a) to be interpreted as the activation of the emotional state of an individual, which is analogous to the excitation of a two-level quantum system in physics. The $\hat{\sigma }^{z}$ specifies emotional arousal variable. In addition, we define Hermitian combinations of $\hat{\sigma }^{\pm }$ as

7a $$\begin{aligned} \hat{\sigma }^{x}&= \hat{\sigma }^{+}+\hat{\sigma }^{-}; \end{aligned}$$

7b $$\begin{aligned} \hat{\sigma }^y&= i(\hat{\sigma }^{-}-\hat{\sigma }^{+}). \end{aligned}$$

The average values for operators in (6), (7) calculated for pure state $|\Psi \rangle$ are

8a $$\begin{aligned}&\sigma ^{x}\equiv \langle \hat{\sigma }^{x} \rangle = \text {sin}\theta \cos \varphi ; \end{aligned}$$

8b $$\begin{aligned}&\quad \sigma ^{y}\equiv \langle \hat{\sigma }^{y} \rangle = - \sin \theta \sin \varphi ; \end{aligned}$$

8c $$\begin{aligned}&\quad \sigma ^{z}\equiv \langle \hat{\sigma }^{z} \rangle = -\cos \theta . \end{aligned}$$

We can recognize $\sigma ^{x,y,z}$ as components of the vector $\vec {\sigma } = (\sigma ^{x}, \sigma ^{y}, \sigma ^{z})$ determined by means of the state $|\Psi \rangle$. These components (for a pure state) lie within the domain $-1\le \sigma ^{x,y,z} \le 1$. In this work we especially interesting in $\sigma ^{z}$ component features. The maximal value, $\sigma ^{z} = 1$, corresponds to the fully activated arousal state, obtained at at $\theta = \pi$ in Fig. 1. The minimal, $\sigma ^{z} = -1$, corresponds to the $\theta = 0$ (inactive, calm state). Below, we focus on the emotional states determined by Eqs. (4) only. The symmetry of the Bloch sphere representation easily allows for recognizing similar results for states of feelings (5). Noteworthy, the transition from any one pair of states in Eqs. (4), (5) to another may be performed by the unitary transformation that practically implies rotations on the Bloch sphere in Fig. 1.

Mathematically, operators $\hat{\sigma }^{x,y,z}$can be established by familiar Pauli matrices, cf.^76^. They provide unitary rotation operators $R_{x,y,z}(\theta )$ with the Bloch vector on the sphere about the *x*, *y* and *z* axes respectively;

9 $$\begin{aligned} R_x(\theta ) = e^{-i\theta \hat{\sigma }^{x}/2}; \quad R_y(\theta ) = e^{-i\theta \hat{\sigma }^{y}/2}; \quad R_z(\theta ) = e^{-i\theta \hat{\sigma }^{z}/2}. \end{aligned}$$

Thus, we can obtain the desired emotional state of individuals by applying rotations on the Bloch sphere. In this case, the emotions of individuals change continuously; the point that specifies emotional state moves on the sphere, see Fig. 1. Notice, the above description of emotional states proposes continuity of changes in emotional states that are discussed in psychology, see e.g.^78^.

Thus, we can associate the Bloch vector properties with the polarization features of the social (affective) vector field by analogy with quantum physics. Operators defined in Eqs. (6), (7) obey the *SU*(2) algebra commutation relations

10a $$\begin{aligned}&[\hat{\sigma }^{z},\hat{\sigma }^{\pm }] = \pm 2\hat{\sigma }^{\pm }; \end{aligned}$$

10b $$\begin{aligned}&\quad [\hat{\sigma }^{+},\hat{\sigma }^{-}] = \hat{\sigma }^{z}; \end{aligned}$$

10c $$\begin{aligned}&\quad [\hat{\sigma }^{x},\hat{\sigma }^{y}] = 2i\hat{\sigma }^{z}; \end{aligned}$$

10d $$\begin{aligned}&\quad [\hat{\sigma }_{0},\hat{\sigma }^{x,y,z}] = 0. \end{aligned}$$

Two additional commutation relations may be obtained from Eq. (10c) by cyclic substitution of the indices.

#### Social tomography of individual’s emotions

Let us discuss how we can measure the emotional states within our approach. At first, it is fruitful to refer to the quantum theory experience. Eq. (10c) implies the Heisenberg uncertainty relation that looks like

11 $$\begin{aligned} \langle (\Delta \hat{\sigma }^{x})^2 \rangle \langle (\Delta \hat{\sigma }^{y})^2 \rangle \ge |\langle \hat{\sigma }^{z}\rangle |, \end{aligned}$$

where $\langle (\Delta \hat{\sigma }^{x,y})^2 \rangle \equiv \langle (\hat{\sigma }^{x,y})^2 \rangle - \langle \hat{\sigma }^{x,y}\rangle ^2$ is the variation of $\sigma ^{x,y}$, which characterizes fluctuations of the corresponding variable. In quantum physics, Eq. (11) manifests the impossibility of measuring the particle spin components in *x* and *y* directions simultaneously with arbitrary high accuracy. In other words, any measurement of one spin component (say, $\sigma ^{y}$) alters the state and evokes uncertainty in another component ($\sigma ^{x}$). Notice, the right side of Eq. (11) is state-dependent. Some trivial case corresponds to the limit $\langle (\Delta \hat{\sigma }^{x,y})^2\rangle =\langle \hat{\sigma }^{z}\rangle = 0$ that may be examined separately. These circumstances open significant tasks and challenges for the measurement of spin components of two-level systems by manipulation of uncertainty relations in quantum optics and atomic physics, e.g.,^75,79–81^.

In general, the specification of the quantum state in physics is based on the quantum tomography procedure, which allows one to obtain information about $\sigma ^{x}$, $\sigma ^{y}$, and $\sigma ^{z}$, e.g.^82,83^. It is important to note that only $\sigma ^{z}$ can be measured directly in a physical experiment; variables $\sigma ^{x}$ and $\sigma ^{y}$ depend on phase $\varphi$and cannot be measured directly in an experiment, cf.^83^. To measure them in quantum optics, it is necessary to explore the other states (3) as a measurement basis that corresponds to some specific rotation operations on the sphere.

We expect that the quantum tomography approach may be useful to fully recognize human emotional states if we associate the Bloch vector properties with human cognitive features. For social systems description, it is useful to account for collective variables for Bloch vector components defined as

12 $$\begin{aligned} P = \sum _{i=1}^N \langle \hat{\sigma }^{-}_i \rangle ; \quad \sigma _{x,y,z} = \sum _{i=1}^N\sigma ^{x,y,z}_i, \end{aligned}$$

where *N* specifies the number of individuals in a social system.

It is worth mentioning that parameter $\sigma _{z}$, often called a social polarization, can be measured in the framework of current social studies. This parameter simply accounts for the social system actors with opposite features, opinions, etc., cf.^84–86^. Noteworthy, in our work we can speak about so-called *affective* social polarization that accounts for opposite emotions and moods within a network social community^87^. The influence of the network community on individuals, as well as the influence of individuals on each other, is the focus of research^2^. The influence of social networks leads to the choice shift and group polarization for small social communities in the networks^23^. In this work, we show how we can measure such a choice shift for individuals when they are communicated through the network.

The measurements of phase-dependent parameters $\sigma ^{x,y}$ are indirect; this can take place due to phase $\varphi _i, i=1,..., N$ variation for the NIAs within the social community. For completely random $\varphi _i$ from (12) we can obtain $P = 0$ which implies $\sigma ^{x,y} = 0$. Thus, for social systems, we should examine parameter *P* in the first place.

In quantum physics, *P* is associated with macroscopic collective polarization of an ensemble of two-level systems. As seen from (6) - (12), parameter *P* is a complex number, $P = |P|e^{i\varphi }$, depending on the phase $\varphi$ distribution. For social study purposes, we can connect *P* with the macroscopic *affective* social field, cf.^66^. In this case, a non-zero *P* that imposes a definite phase and enables characterization of coherent features of the social system as some manifestation of social groups’ cohesion, cf.^88^.

An approach discussed above enables to elucidate the properties of *P*; we should perform two different measurements within the social system, choosing other basic states (3). So, if we recast operators $\hat{\sigma }^{x,y}$ given in (7) through states (3) we obtain

13a $$\begin{aligned} \hat{\sigma }^{x}&= |+\rangle \langle +| - |-\rangle \langle -|\equiv \hat{n}^{(+)} - \hat{n}^{(-)}; \end{aligned}$$

13b $$\begin{aligned} \hat{\sigma }^{y}&= |-i\rangle \langle -i| - |+i\rangle \langle +i| \equiv \hat{n}^{(-i)} - \hat{n}^{(+i)}. \end{aligned}$$

Notice, Eqs. (13) imply practical instruction on how we can measure the social field *P*.

First, we need to change the basis of our system to (3a), which corresponds to emotional states (5b). It is necessary to measure the total average numbers of agents with opposite features $\sum _{j=1}^N\langle \hat{n}_j^{(+)}\rangle$ and $\sum _{j=1}^N\langle \hat{n}_j^{(-)}\rangle$, respectively.

Second, we change the basis to (3b), which corresponds to emotional states (5c), and measure again the total average numbers of agents with opposite features $\sum _{j=1}^N\langle \hat{n}_j^{(+i)}\rangle$ and $\sum _{j=1}^N\langle \hat{n}_j^{(-i)}\rangle$, respectively. The social field phase properties may be inferred from these measurements.

Noteworthy, we can also recognize *P*as some analogue of the wave function that we ascribe social system. Currently, in cognitive science, a quantum-like approach is used for characterization of some non-conventional effects, e.g.,^34,35^. In this case, a certain phase of the wave function determines a quantum-like interference phenomenon that is inherent to DM processes performed under uncertainty^89^. Below, we show that non-zero values of the *P*-parameter specify a non-equilibrium phase transition that occurs in the DIS and accounts for complex network peculiarities.

### Open DIS model

#### DIS network

**Fig. 2:**
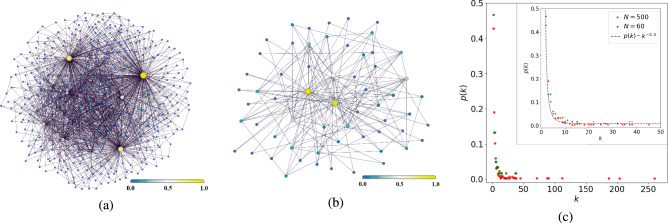
(**a**, **b**) AIA – AIA scale-free networks colored according to the node eigenvector centrality and (c) their node degree distributions. The parameters are: $k_{min} = 2$, $k_{max} = 260$, $\langle k\rangle \simeq 6.8$, $\zeta \simeq 30.8$, $N=500$ for (**a**), and $k_{min} = 2$, $k_{max} = 38$, $\langle k\rangle \simeq 5.2$, $\zeta \simeq 14.6$, $N=60$ for (**b**), respectively. Inset demonstrates approximation of these networks by the power node degree distribution (14) with degree exponent $\nu \simeq 2.3$.

The DIS model we consider in this work is shown in Fig. 2. We plot AIA – AIA network as an undirected graph that approaches a power law degree distribution (PLDD)

14 $$\begin{aligned} p(k) = \frac{\mathcal {N}}{k^{\nu }}, \end{aligned}$$

where *k* is the node degree; $\nu>1$ is the degree exponent; $\mathcal {N}$ is the normalization factor that is determined by the minimal node degree and $\nu$, see^20^.

In Fig. 2(a, b), we demonstrate two complex (scale-free) graphs generated by the **igraph** library of the R language and then processed via Python **NetworkX**. In Fig. 2(c), we plot the distribution of node degrees $p_k$. Noteworthy, both of the graphs approach degree distribution *p*(*k*) in a continuous limit with degree exponent $\nu = 2.3$, see the inset in Fig. 2(c). The smallest graph in Fig. 2(b) is more suitable for our numerical calculations performed in this work due to computational resource saving, and all the results obtained are scalable to the bigger graph in Fig. 2(a).

The statistical properties of the network that we are interested in here are determined as

15 $$\begin{aligned} \langle k\rangle =\frac{1}{N}\sum _{i=1}^{N} k_i; \quad \zeta \equiv \frac{1}{N\langle k\rangle }\sum _{i=1}^N {k_i^2}, \end{aligned}$$

where $k_{i}$ is the *i*-th node degree, $\langle k\rangle$ is the average node degree, $\zeta$ characterizes the normalized second moment of the node degree; the key parameters and variables used in this work are displayed in Table 1. We can express $\langle k\rangle = \frac{1}{N}\sum _{i,j}^{N} A_{ij}$ via symmetric adjacency matrix $A_{ij}$ that determines topological properties of the network in Fig. 2: for any $i,j = 1,...,N$, $A_{ij}=1$ if two *i* and *j* nodes are linked and $A_{ij} = 0$ otherwise. In addition, we suppose that loops are inadmissible for network nodes, $A_{ii} = 0$. Statistical properties for the networks in Fig. 2(a,b) clearly demonstrate the hubs, which are the nodes with maximal node degree^39^.

**Table 1:** List of basic parameters and variables.

Symbol	Meaning
$A_{ij}$	Element of the$N\times N$symmetric adjacency matrix that determines topological properties of the DIS network
$\langle k\rangle$	Average node degree
$\zeta$	Normalized second moment of the node degree
$g_i=g$	NIA – AIA interaction strength
*J*	AIA – AIA coupling strength
$\Omega _{i}$	s-photon energy provided by the *i*-th AIA
$\Omega _{0,i}$	Social energy of the *i*-th NIA (user) activation
$\Delta _i=\Omega _{i}-\Omega _{0,i}$	Social energy detuning that defines how *i*-th NIA susceptible to information provided by the AIA
$\Gamma _i$	Deactivation rate of the *i*-th NIA emotional state
$\gamma _{e,i}$	Rate of spontaneous change of the *i*-th NIA’s emotions and moods
$\gamma _{p,i}$	External information pump rate for *i*-th NIA
$\kappa _i=\kappa$	Information loss rate
$\alpha _i; E_i =\alpha _i e^{i\Omega _{0,i}t}$	Complex variables that specify the amplitude of the average coherent information field established by the *i*-th AIA
$\sigma _i^+=(\sigma _i^-)^*$	Complex variable that specify that average emotional activation (excitation) of the *i*-th user
$\sigma _i^z$	Average emotional state of the *i*-th NIA (arousal) experienced in response to the information provided by their AIA
$\sigma _{0,i}^z$	Steady-state arousal of the *i*-th NIA
$\sigma ^{x,y,z}_i$	Components of the *i*-th NIA affective vector $\vec {\sigma }_i = (\sigma ^{x}_i, \sigma ^{y}_i, \sigma ^{z}_i)$ associated with the affective sphere
$R_i$	Affective sphere radius of the *i*-th NIA
$F^{x,y}_i, F_i$	Forces acting on the *i*-th NIA from the other network actors

#### Quantum-like model of DIS

**Fig. 3:**
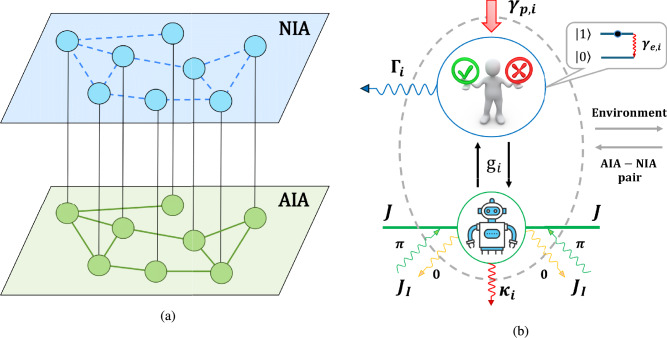
(**a**) Two-layer $\texttt {AIA -- NIA}$ network of a collaborative multi-agent system consisting of NIA – AIA pairs. Blue circles characterize the NIAs that interact only with their AIAs (green circles) which communicate via the AIA – AIA graph. Blue dashed lines (**b**) - Sketch of the *i*-th pair of NIA (human in a blue circle) and their avatar (robot picture in a green circle). $g_i$ is the AIA – NIA coupling parameter that indicates the frequency of the *i*-th avatar – user communication. NIA can obtain information from the environment with a rate $\gamma _{p,i}$; $\Gamma _i$ is their deactivation (dephasing) rate. *J* is the AIA – AIA coupling strength; we assume that information non-homogeneously transmits within the AIA – AIA network and can be lost (the 0-phase) or enhanced by it (the $\pi$-phase) with the rate $J_I$; $\kappa _i$ is the losses that the *i*-th AIA admits. Other details are given in the text.

We assume that DIS supports a collaborative multi-agent system represented as a two-layer network in Fig. 3(a), cf.^90,91^. In particular, our DIS contains two types of agents: the upper, blue layer $\texttt {NIA}$ in Fig. 3(a) establishes uncoupled NIAs (humans), while the green layer $\texttt {AIA}$ in Fig. 3(a) corresponds to the AIAs (avatars or LLM-based agents) connected through a complex network shown in Fig. 2(a). According to the model in Fig. 3(a), AIAs can disseminate information within the network and communicate with each other and with users simultaneously. To be more specific, we assume that NIAs interact with their AIAs only, see vertical lines in Fig. 3(a). In practice, several NIAs can use the same NIA, so the number of AIAs may be less than NIAs. In this case, we assume that the numbers of AIAs and NIAs are equal, but some AIAs may be identical. Thus, each of the *N* network nodes in Fig. 2(a) establishes exactly one AIA – NIA pair.

In this work, we assume that NIAs cannot communicate with each other directly, cf.^30^. However, the communication between AIA within the DIS promotes establishing social links that we depicted by using blue dashed lines at the upper layer $\texttt {NIA}$. Since NIAs do not communicate with each other directly, such links reflect moods, emotional relationships between NIAs within the DIS. Therefore, in this work, our primary focus is to identify the emotions and moods mediated by AI agents’ interactions.

We describe NIA – AIA interaction by an analogy with our quantum-like model provided in previous work^30^; here we only briefly establish its key properties. In Fig. 3(b), we demonstrate an arbitrary NIA – AIA pair as an open system within the network. We assume that information within the network is transferred through so-called s-photons, which represent meaningful information for NIAs and can be recognized as short messages, notices, etc.^28^. AIAs recommend to users the information received from the AIA – AIA network; users can accept or reject this information. NIAs cannot communicate with each other directly, but they can obtain information from the environment, i.e., from the outside of a network community, with rate $\gamma _{p,i}$, Fig. 3. Thus, the emotional state of the users changes due to interaction with their LLM-based agents and environment. Notice, the quantum-like description of NIA’s emotional states presumes the existence of a spontaneous change of mood. Typically, humans spend a limited time $\tau ^e_i$ in the emotionally excited state. In Fig. 3, the parameter $\gamma _{e,i}\simeq 1/\tau ^e_i$ specifies spontaneous transition from state $|1\rangle _i$, which is more energetic, accounting for our specifications in Eqs. (4), (5).

We represent the DIS model considered in this work in terms of the same Hamiltonian $\hat{H}$ as we discussed in^30^:

16 $$\begin{aligned} \hat{H} = \hbar \sum _{i=1}^{N}{\Bigg [\frac{1}{2}\Omega _{0,i}\hat{\sigma }_i^z + \Omega _i\hat{a}_i^{\dag }\hat{a}_i+g_i(\hat{a}_i\hat{\sigma }_i^+ + \hat{a}_i^{\dag }\hat{\sigma }_i^-)\Bigg ]} -\frac{\hbar J}{2}\sum _{i=1}^{N}\sum _{j=1}^{N}{A_{ij}(\hat{a}_i^{\dag }\hat{a}_j + \hat{a}_j^{\dag }\hat{a}_i)}. \end{aligned}$$

Hamiltonian (16) is established by the Hermitian operator $\hat{H}$ that describes the main coherent processes that occur in DIS. In physics, a Hamiltonian corresponds to the average energy of the entire system. In social and economic sciences, it is related to the cost function. In fact, the mean-field dynamic equations and their solutions that we are going to obtain for the variables characterizing agents correspond to the solution of the optimization problem using quantum theory methods.

The terms in the square brackets in (16) characterize elementary processes that occur with NIA in the presence of their interaction with AIAs (the last term in these brackets). We describe the number of s-photons by $\hat{a}_i^{\dag }\hat{a}_i$ operator relevant to the *i*-th AIA. S-photons are able to transfer user moods, which we take into account by the social energy $\hbar \Omega _i$. In the framework of a quantum-like approach, we specify the s-information the *i*-th AIA provides to its user by annihilation (creation) $\hat{a}_i$ ($\hat{a}_i^{\dag }$) operators that obey bosonic commutation relations

17 $$\begin{aligned} [\hat{a}_i, \hat{a}^{\dag }_j] = \delta _{ij}, \quad i, j=1,...,N. \end{aligned}$$

In (16), the third term is relevant to the *i*-th user – AIA interaction characterized by strength $g_i$; thereafter, we suppose that $g_i = g$ for all AIA – NIA pairs in DIS. In particular, s-photon absorption evokes (emotional) activation of individuals; the operators $\hat{\sigma }^{-}_i$, $\hat{\sigma }^{+}_i$ that are relevant to the *i*-th user are specified in (6). Hereafter, we also set the Planck constant $\hbar =1$. The last term in (16) is relevant to the AIAs’ coupling; it specifies AIA – AIA communication and information exchange within the network, which we characterize by parameter *J*, see also Table 1.

#### Mean-field equations

Let us first assume for this moment that parameter *J* is real, which corresponds to a Hermitian Hamiltonian (16). We use the Heisenberg-Langevin approach (see, e.g.,^92^) for obtaining basic equations that read as

18a $$\begin{aligned}&\dot{\hat{a}}_i = -(i\Omega _{i} + \kappa _{i})\hat{a}_i - ig\hat{\sigma }^-_i + iJ\sum _{j=1}^N A_{ij}\hat{a}_j + \hat{F}_{ph,i}; \end{aligned}$$

18b $$\begin{aligned}&\quad \dot{\hat{\sigma }}^-_i = -(i\Omega _{0,i} + \Gamma _i)\hat{\sigma }^-_i + ig\hat{\sigma }_i^z \hat{a}_i + \hat{F}_{-,i}; \end{aligned}$$

18c $$\begin{aligned}&\quad \dot{\hat{\sigma }}_i^z = (\gamma _{p,i} - \gamma _{e,i}) - (\gamma _{p,i} + \gamma _{e,i})\hat{\sigma }_i^z + 2ig(\hat{a}_i^{\dag }\hat{\sigma }^-_i - \hat{a}_i\hat{\sigma }^+_i) + \hat{F}_{z,i}, \end{aligned}$$

where the following notations are introduced: $\Gamma _i$ is the rate of attenuation of NIA’s aroused emotional state; $\kappa _i$ is the information loss rate by the *i*-th AIA; $\gamma _{p,i}$ is the pumping rate for information that users can obtain from the environment; $\gamma _{e,i}$ is the rate that corresponds to the spontaneous change of NIA’s moods. In (18), we account for the Langevin force operators $\hat{F}_{ph,i}$, $\hat{F}_{-,i}$, and $\hat{F}_{z,i}$; consideration of quantum-like effects allows establishing operators in (18) in the form

19a $$\begin{aligned}&\hat{\sigma }^{\pm }_i \simeq \langle \hat{\sigma }^{\pm }_i\rangle + \delta \hat{\sigma }^{\pm }_i \equiv \sigma _i^{\pm } + \delta \hat{\sigma }^{\pm }_i; \end{aligned}$$

19b $$\begin{aligned}&\quad \hat{\sigma }_i^z \simeq \langle \hat{\sigma }_i^z\rangle + \delta \hat{\sigma }_i^z \equiv \delta \hat{\sigma }^{z}_i + \delta \hat{\sigma }_i^z; \end{aligned}$$

19c $$\begin{aligned}&\quad \hat{a}_i\simeq \langle \hat{a}_i\rangle + \delta \hat{a}_i \equiv \alpha _i + \delta \hat{a}_i, \end{aligned}$$

where $\alpha _i = \langle \hat{a}_i\rangle$ is the complex amplitude of the average coherent information field established by the *i*-th AIA within the DIS; $\sigma _i^+ = \langle \hat{\sigma }^+_i\rangle$ and $\sigma _i^- = \langle \hat{\sigma }^-_i\rangle$ are the expectation values describing activation and deactivation of the *i*-th user’s emotional state, cf. Table 1. The $\sigma ^z_i = \langle \hat{\sigma }_i^z\rangle$ specifies the average emotional state of the user (average arousal), which emerges as a result of the emotional response to the information provided by their AIA, $i = 1,2,..., N$, cf.^28,31^.

Operators $\delta \hat{\sigma }^{\pm }_i, \, \delta \hat{\sigma }_i^z, \, \delta \hat{a}_i$ in (19) characterize small fluctuations in respect of average values $\langle \hat{\sigma }^{\pm }_i\rangle ,\, \langle \hat{\sigma }_i^z\rangle , \, \langle \hat{a}_i\rangle$, respectively, cf.^92^. Within the mean-field approach, it is possible to neglect quantum fluctuations in (19), as well as correlations of Langevin force operators. In this limit, we are able to replace operators $\hat{\sigma }_i^z$, $\hat{a }_i, \hat{a}^{\dag }_i, \hat{\sigma }^{\pm }_i$ in Eqs. (19) by their averages, which, in fact, represent classical *C*-numbers. This approach is justified in the case of open (driven-dissipative) DIS, which has a large number of agents. Notice, the averages of the Langevin force operators are $\langle \hat{F}_{ph,i}\rangle = \langle \hat{F}_{-,i}\rangle = \langle \hat{F}_{z,i}\rangle = 0$. As a result, from (19) we obtain

20a $$\begin{aligned} \dot{\alpha }_i&= (-i\Omega _i -\kappa _i)\alpha _i - ig \sigma _i^- + iJ\sum _{j=1}^{N}{A_{ij}\alpha _j}; \end{aligned}$$

20b $$\begin{aligned} \dot{\sigma }_i^-&= (-i\Omega _{0,i} -\Gamma _i)\sigma _i^- + ig\sigma _i^z\alpha _i; \end{aligned}$$

20c $$\begin{aligned} \dot{\sigma }_i^z&= (\gamma _{p,i} -\gamma _{e,i}) -(\gamma _{p,i} + \gamma _{e,i})\sigma _i^z + 2ig(\alpha _i^*\sigma _i^- - \alpha _i\sigma _i^+). \end{aligned}$$

Then, we remove rapidly oscillating components in Eq. (20): substituting $\sigma _i^-(t) = p_ie^{-i\Omega _{0,i}t},\, \alpha _i(t) = E_ie^{-i\Omega _{0,i}t}$ into Eq. (20) we obtain

21a $$\begin{aligned} \dot{E}_i&= (-i\Delta _i - \kappa _i)E_i - igp_i + iJ\sum _{j=1}^{N}{A_{ij}E_j}; \end{aligned}$$

21b $$\begin{aligned} \dot{p}_i&= -\Gamma _ip_i + ig\sigma ^z_iE_i; \end{aligned}$$

21c $$\begin{aligned} \dot{\sigma }^z_i&= (\sigma ^z_{0,i} - \sigma _{i}^z)(\gamma _{p,i} + \gamma _{e,i}) + 2ig(E_i^*p_i - E_ip_i^*), \end{aligned}$$

where we define (cf. Table 1)

22 $$\begin{aligned} \sigma ^z_{0,i} = \frac{\gamma _{p,i} - \gamma _{e,i}}{\gamma _{p,i} + \gamma _{e,i}}. \end{aligned}$$

In Eqs. (21) $\Delta _i \equiv \Omega _i - \Omega _{0,i}$ is the detuning. From a practical point of view, $\Delta _i$ specifies how individuals are susceptible to network-enforced information provided by the AIAs, and how relevant it is to the user’s vital needs. Obviously, the limit $\Delta _i = 0$ characterizes a resonant condition that implies maximal activation of the users. Eq. (22) plays an important role for our purposes. In particular, at $\sigma ^z_{0,i}> 0$ user obtains a large amount of information from external mass media, the information pumping rate is high enough, and $\gamma _{p,i}> \gamma _{e,i}$. Obviously, in this limit, we can propose that the user becomes emotionally excited, occupying the upper half of the sphere in Fig. 12. At $\gamma _{p,i} = \gamma _{e,i}$ from (22) we obtain $\sigma ^z_{0,i} = 0$ that corresponds to an emotionally neutral state. In this case, the activation evoked by eternal mass media is compensated (on average) by the user’s emotional attenuation rate. The limit $\sigma ^z_{0,i} < 0$ is not interesting for us in this work since we consider here only NIAs in emotionally excited states.

Note that within the framework of the quantum-like approach to modeling NIA, the parameters $\sigma ^z_{0,i}, \gamma _{e,i}, \Delta _i, \Gamma _i, g_i$, $i=1,...,N$, specify various properties that determine the homogeneity of an NIA community. These properties can be associated with the agents’ cognitive characteristics, their attitude towards (or perception of) external information (parameters $\sigma ^z_{0,i}, \Delta _i$), communicative ability (parameter $g_i$, which we consider in this work as $g_i=g$) DM rationality (parameter $\gamma _{e,i}$), emotional intelligence, and other traits. In real-world settings, the inevitable heterogeneity of users can serve as a source of diverse social phenomena in networked communities, cf.^93–95^. In our context, heterogeneity refers to variation in the specified parameters within certain bounds. In previous work^30^, we examined how the variability of parameters $\Delta _i$ and $\Gamma _i$ affects the emergence of collective opinions and self-organization in DIS. In^30^, we also introduced the NIA cooperativity parameter, which accounts for a combination of parameters $\kappa _i, \Gamma _i, g$, and determines the degree of agents’ cooperation within the DIS. In this study, we focus on near-homogeneous NIA communities, where the parameters under consideration differ only slightly, or this difference can be neglected. This approach enables the identification of trends in the emotional behavior of the community by distinguishing individual trajectories within the affective sphere. In contrast, when the agent system exhibits substantial heterogeneity, simulations reveal a significant increase in the complexity of emotional states. Addressing such systems requires a separate analysis.

The approach that we use below is based on adiabatic elimination of the s-photon field, leaving NIAs’ emotional state variables. In this case, the set of parameters $\kappa _i$, $i = 1,...,N$, requires clarification. One may assume that the lifetime of the message provided by the *i*-th LLM-agent to its user is determined by the value of the parameter $\tau _i\simeq 1/\kappa _i$, cf.^28^. In practical terms, this lifetime reflects the degree to which the *i*-th AIA’s message is meaningful to the corresponding NIA, thereby influencing its DM process. Although LLM agents are capable of maintaining conversations, their messages to users are typically informative (and potentially relevant) rather than decisive. In those rare cases where a message has a lasting impact, its lifetime would be relatively long ($\kappa _i$ small), and it could be further amplified within the echo-chambers surrounding the user. Unlike conventional chatbots, which are pre-programmed to achieve specific communication goals, modern LLM-agents generate diverse messages that allow them to adapt dynamically to users.

Strictly speaking, LLM-based agents predict the next token according to statistical patterns in training data rather than the objective truth. A hallucination arises when the predicted continuation, though highly probable, diverges from factual reality. In particular, a single message corresponding to a non-zero value of $\kappa _i$ may indicate that the AIA (using its LLM algorithms) did not correctly determine the relevant contents (or the user’s emotional components) in the text message. The lifetime of such a message will be extremely short, since it will require new “iterations” in the dialogue with the user. However, in the course of message exchange, such an agent is capable of adapting to the user. As a result, LLM-agents tend to “adiabatically” follow the broader discourse and tonality of conversations with NIAs, thereby demonstrating their supportive role. In this adiabatic approximation, one may assume that all $\kappa _i$ are very large, and for further simplification of Eqs. (21) we use the following inequality chain

23 $$\begin{aligned} \kappa _i \gg \Gamma _i, \gamma _{e,i}, g> |J|. \end{aligned}$$

First condition in (23) specifies adiabatic approximation that is typically used in quantum physics in the framework of steady-state superradiant laser investigations, cf.^96^. Second, inequality in (23) means relatively weak coupling between the AIAs in the network. Due to the condition (23) below, we neglect the variations of $\kappa _i$, assuming $\kappa _i \simeq \kappa$ for all *i*s. In this limit, we can adiabatically remove $E_i$ from Eqs. (21) assuming $\dot{E}_i = 0$. In the zeroth-order approximation from (21a) we obtain

24 $$\begin{aligned} E_i^{(0)} = -\frac{gp_i}{\Delta _i - i\kappa } = - \frac{gp_i(\Delta _i + i\kappa )}{\Delta _i^2 + \kappa ^2}. \end{aligned}$$

The Eq. (24) admits a simple explanation. In the zeroth-order approximation, we can neglect the network influence effect on NIA and consider an AIA– NIA pair separately. Noteworthy, users are able to obtain information from the environment, which changes this emotional state, determined in (24) by affective field $p_i$. In this limit, (24) means that AIAs simply follow users’ affective field.

In the presence of a network, situation changes. Substituting (24) into Eqs. (21a) and assuming $\dot{E}_i = 0$, we obtain

25 $$\begin{aligned} E_i = E_i^{(0)} - iJg\sum _{j=1}^{N}{A_{ij}p_j\frac{\Delta _j + i\kappa }{\Delta _j^2 + \kappa ^2}} \end{aligned}$$

Eq. (25) plays a significant role in this work. In particular, (25) specifies the AIA – AIA field established in the network. The emotional state of the *i*-th AIA agent simply follows the NIAs’ social field, which is shaped by the perturbations $p_i\simeq\sigma _i^-$ (see (24)), and further modulated by their arousal level to such perturbations (parameters $\sigma _{z,i}$), as shown below in Eq. (26), cf.^42,43^. Thus, condition (25) allows adiabatically removing this field and focusing on the user’s mental and emotional features in DIS. Notably, last term in (25) is small in comparison with $E_i^{(0)}$ due to the last inequality in (23).

Substituting (25) into (21b) we immediately arrive at coupled equations

26a $$\begin{aligned} \dot{p}_i&= -\left( \Gamma _i + i\sigma ^z_{i}\left( D_i + iC_i\right) \right) p_i {- \frac{iJ\sigma ^z_i}{g^2}\sum _{j=1}^{N}A_{ij}p_j\left( D_i + iC_i\right) \left( D_j + iC_j\right) ;} \end{aligned}$$

26b $$\begin{aligned} \dot{\sigma }^z_i&=(\sigma ^z_{0,i}-\sigma _{i}^z)\gamma _{+,i}- 4C_i|p_i|^2, \end{aligned}$$

where $\gamma _{+,i} \equiv \gamma _{p,i} + \gamma _{e,i}$. The term proportional to $|p_i|^2$ in (26b) characterizes the nonlinear self-activation effects of individuals’ emotions; we suppose them to be weak enough. In (26), we made definitions

27 $$\begin{aligned} C_i\equiv \frac{\kappa g^2}{\Delta _i^2+\kappa ^2}; \quad D_i\equiv \frac{\Delta _ig^2}{\Delta _i^2+\kappa ^2} = \tilde{\Delta }_i C_i, \end{aligned}$$

where $$\tilde{\Delta }_i\equiv \Delta _i/\kappa$$.

The combination of parameters $\mathcal {C}_i\equiv C_i/\Gamma _i$ (see (27)) specifies the *i*-th user cooperativity parameter; it determines NIA’s cooperativity abilities in the network, which is realized through AIA’s. At $\Delta _i = 0$, parameter $C_i$ takes on a familiar look, which we discussed in^30^. We examine the features of $\mathcal {C}_i$ for our problem in the next subsection.

Unlike our previous work^30^, now we recognize parameter *J* as a complex:

28 $$\begin{aligned} J = J _ R + i\cos \Theta J_I\equiv J_R + iJ_{I,eff}. \end{aligned}$$

Here, $J_R$ specifies the coherent information transfer between the AIAs, while $J_I$ determines diffusive (incoherent) couplings; $\Theta$ is the phase that characterizes reservoir-network interaction and specifies non-uniform losses within the network. We identify the limit $\Theta = 0$ as the 0-phase regime that corresponds to losses of information immediately within the network. On the contrary, at $\Theta =\pi$ we obtain the $\pi$-phase regime that relates also to the active complex networks, see Fig. 3.

We can rewrite Eqs. (26) in terms of real variables $\sigma ^{x,y,z}_i$ for the *i*-th NIA in the form

29a $$\begin{aligned} \dot{\sigma }^x_i&= -\Gamma _i \sigma ^x_i+\sigma ^z_{i}(C_i\sigma ^x_i -D_i\sigma ^y_i )+F^x_i; \end{aligned}$$

29b $$\begin{aligned} \dot{\sigma }^y_i&= -\Gamma _i \sigma ^y_i +\sigma ^z_{i}(C_i\sigma ^y_i + D_i\sigma ^x_i )+F^y_i; \end{aligned}$$

29c $$\begin{aligned} \dot{\sigma }^z_i&=(\sigma ^z_{0,i}-\sigma _{i}^z)\gamma _{+,i}- C_i\Big ((\sigma ^x_i)^2+(\sigma ^y_i)^2\Big ); \end{aligned}$$

29d $$\begin{aligned} R_i\dot{R}_i&= - \Gamma _i R_i^2 + (\Gamma _i - \gamma _{+,i})(\sigma ^z_i)^2 + \gamma _{+,i}\sigma ^z_{0,i} \sigma ^z_i, \end{aligned}$$

where we introduce the variables

30a $$\begin{aligned} \sigma ^{\perp }_i&= \sqrt{(\sigma ^x_i)^2 + (\sigma ^y_i)^2}; \end{aligned}$$

30b $$\begin{aligned} R_i&= \sqrt{(\sigma ^z_i)^2 + (\sigma ^{\perp }_i)^2}. \end{aligned}$$

In Eqs. (29) we introduced

31 $$\begin{aligned} F^{x,y}_i = \frac{\sigma ^z_{i}}{g^2}\Big ( (D^2-C^2)J_{I,eff} +2CD J_R\Big )\sum _{j=1}^{N}A_{ij}\sigma _j^{x,y} \mp \frac{\sigma ^z_{i}}{g^2}\Big ((D^2-C^2)J_R - 2CD J_{I,eff}\Big )\sum _{j=1}^{N}A_{ij}\sigma _j^{y,x}, \end{aligned}$$

which specify forces that act from the network community on the *i*-th NIA. In (31), we assume for simplicity that $\Delta _i$ is the same for all *i*-th and thus $C_i \equiv C$, $D_i \equiv D$.

**Fig. 4:**
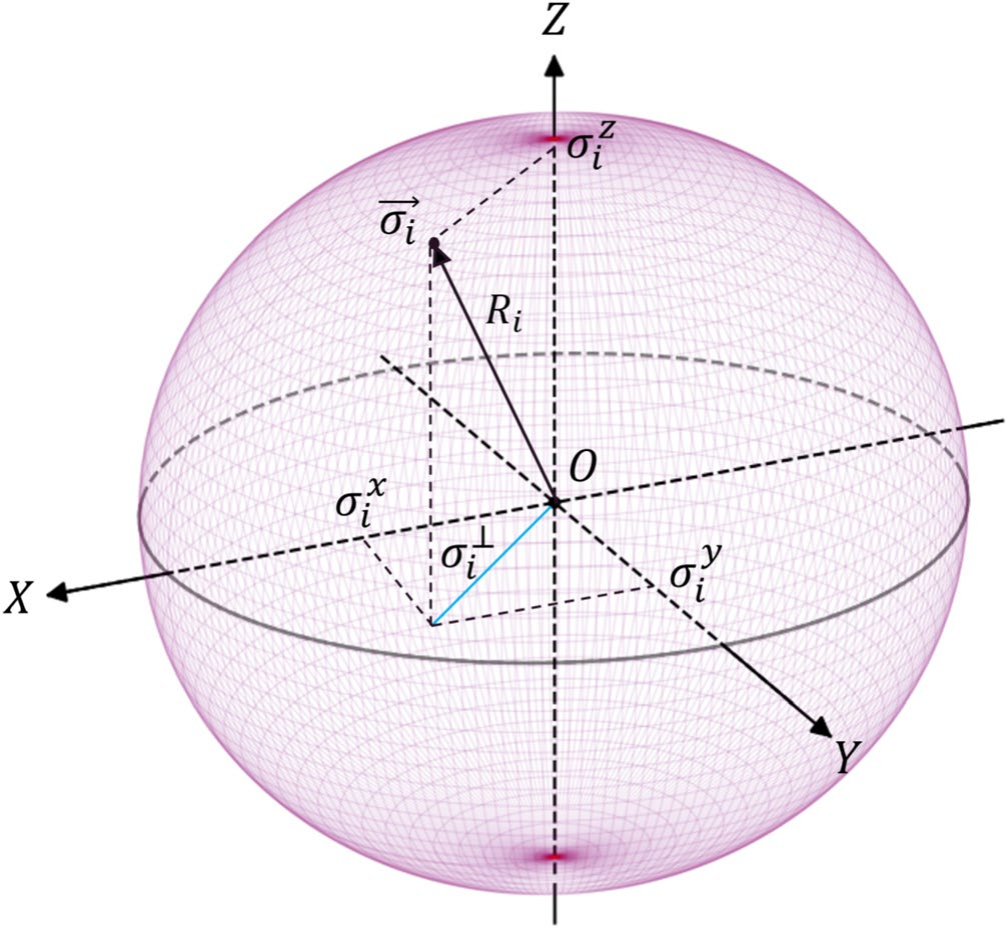
Affective sphere representation for specification of the *i*-th user emotional states in DIS. $R_i$ is the length of the affective vector $\vec {\sigma }_i$; $\sigma ^{\perp }_i$ is a projection of the affective vector onto the *XOY* plane. The details are explained in the text.

Thus, a set of Eqs. (29) characterizes open driven-dissipative DIS for the mean-field (classical) variables $\sigma ^{x,y,z}_i$, $R_i$ in the presence of network-inspired external forces. In Fig. 4 we give the *affective sphere* representation as a tool for geometrical interpretation of variables $\sigma ^{x,y,z}_i$, $\sigma ^{\perp }_i$ specified for the *i*-th individual in (29). In particular, $\sigma ^{x,y,z}_i$ are components of their affective vector $\vec {\sigma }_i = (\sigma ^{x}_i, \sigma ^{y}_i, \sigma ^{z}_i)$ that possesses radius $R_i$, see Eg. (30b) and Table 1. $\sigma ^{\perp }_i$ represents the projection of the affective vector onto the *XOY* plane and characterizes the combination of emotions for a given level of an individual’s activation (arousal). Noteworthy, since $R_i$ relates to the emotional states of different individuals operating within driven-dissipative DIS, it is fruitful to leave $R_i$ non-normalized. In particular, $R_i$ represents the length of affective vector $\vec {\sigma }_i$ and specifies how intensive the emotional state of the *i*-th user is.

## Results

### Network-free individuals emotional states, $J=0$

#### Emotional states at steady-state

Let us examine the *i*-th NIA’s basic features without network coupling, $J=0$ that implies $F^x_i = F^y_i = 0$ in (29). At $\dot{\sigma }^{x,y,z}_i = 0$ from (29) we obtain

32a $$\begin{aligned} 0&= -\Gamma _i \sigma ^x_i+\sigma ^z_{i}(C_i\sigma ^x_i -D_i\sigma ^y_i); \end{aligned}$$

32b $$\begin{aligned} 0&= -\Gamma _i \sigma ^y_i +\sigma ^z_{i}(C_i\sigma ^y_i + D_i\sigma ^x_i); \end{aligned}$$

32c $$\begin{aligned} 0&= (\sigma ^z_{0,i} - \sigma _{i}^z)\gamma _{+,i} - C_i\Big ((\sigma ^x_i)^2 + (\sigma ^y_i)^2\Big ). \end{aligned}$$

The trivial solution of Eqs. (26), (29) is

33 $$\begin{aligned} \sigma ^z_{0,i} = \sigma ^{z}_i; \quad \sigma ^{x}_i = \sigma ^{y}_i = 0. \end{aligned}$$

Eqs. (32a), (32b) lead to equation

34 $$\begin{aligned} (1 + \tilde{\Delta }_i^2)C_i^2(\sigma _{i}^z)^2 - 2\Gamma _iC_i\sigma ^z_{i} + \Gamma _i^2 = 0, \end{aligned}$$

which implies a non-trivial solution of (32) at $\Delta _i=0$ and specifies critical arousal $\sigma ^z_{cr,i}$ in the form

35 $$\begin{aligned} \sigma ^z_{cr,i} = \frac{\Gamma _i }{C_i} = \frac{\Gamma _i \kappa }{g^2}. \end{aligned}$$

In this case, we are able to examine the real affective field $p_i = |p_i|$ that we establish as

36 $$\begin{aligned} |p_i| \equiv \frac{1}{2}\sigma ^{\perp }_i. \end{aligned}$$

At $\Delta _i = 0$ non-trivial solutions of (32) imply

37 $$\begin{aligned} \sigma ^{\perp }_i = \sigma ^{\perp }_{0,i}\sqrt{\frac{\sigma ^z_{0,i}}{\sigma ^z_{cr,i}} - 1}; \end{aligned}$$

where $\sigma ^z_{cr,i}$ is given in (35); $\sigma ^{\perp }_{0,i} = \sqrt{\gamma _{+,i}\sigma ^z_{cr,i}/C_i}$. Thus, in this limit, the affective field (variable $\sigma ^{\perp }_i$) exhibits critical behavior. As far as $\sigma ^z_{0,i}\le \sigma ^z_{cr,i}$, variable $\sigma ^{\perp }_i=0$ and the steady-state is the north pole of the affective sphere with $\sigma ^z_i = 1$. In the vicinity of critical point $\sigma ^z_{0,i} \simeq \sigma ^z_{cr,i}$ from (41) follows the affective sphere critical radius

38 $$\begin{aligned} R_i \simeq |\sigma ^z_{cr,i}| \le 1. \end{aligned}$$

At $\sigma ^z_{0,i}> \sigma ^z_{cr,i}$ $\sigma ^{\perp }_i\ne 0$. Solution (37) approaches the ring on the affective sphere in a plane perpendicular to the axis $\sigma _i^z$. Condition $R_i = 1$, which implies

39 $$\begin{aligned} \Gamma _i^2 + \gamma _{+,i} (C_i\sigma ^z_{0,i} - \Gamma _i) = C_i^2, \end{aligned}$$

allows to define the criteria when the affective sphere radius grows ($R_i>1$) or vanishes ($0<R_i<1$). It depends on the ratio $\tilde{\gamma }^+_i \equiv \gamma _{+,i}/\Gamma _i$, which largely depends on the psychological characteristics of individuals. When

40 $$\begin{aligned} \frac{\gamma _{+,i}}{\Gamma _i}> \frac{1}{\sigma _{cr,i}^z}\frac{1 - (\sigma _{cr,i}^z)^2}{\sigma _{0,i}^z - \sigma _{cr,i}^z} \end{aligned}$$

the sphere grows, otherwise it shrinks. Here it is also appropriate to mention the effect of mental inflation, which has been known for a long time^97^.

#### Emotional state dynamics

To describe emotional state dynamics at $J = 0$, it is fruitful to rewrite (29) in variables $\sigma ^{\perp }_i$ and $\sigma ^{z}_i$ as

41a $$\begin{aligned}&\sigma ^{\perp }_i\dot{\sigma }^{\perp }_i = (\sigma ^{\perp }_i)^2 (C_i\sigma ^z_i - \Gamma _i); \end{aligned}$$

41b $$\begin{aligned}&\quad \dot{\sigma }^z_i = - (\sigma ^z_{0,i} - \sigma ^z_i)\gamma _{+,i} -C_i(\sigma ^{\perp }_i)^2; \end{aligned}$$

41c $$\begin{aligned}&\quad R_i\dot{R}_i = - \Gamma _iR_i^2 + (\Gamma _i - \gamma _{+,i})(\sigma ^z_i)^2 + \gamma _{+,i}\sigma ^z_{0,i}\sigma ^z_i. \end{aligned}$$

Set of Eqs. (41) admits a simple analytical solution in the limit of $\gamma _{+,i} \simeq 0$, $\Gamma _{i} = 0$. From (41) we obtain

42a $$\begin{aligned} \sigma ^{\perp }_i&= \text {sech} \left( C_i(t - t_d)\right) ; \end{aligned}$$

42b $$\begin{aligned} \sigma ^z_i&= - \tanh \left( C_i(t - t_d)\right) ; \end{aligned}$$

42c $$\begin{aligned} R_i&= R_i(0) = 1, \end{aligned}$$

where $t_d$ is specified below.

**Fig. 5:**
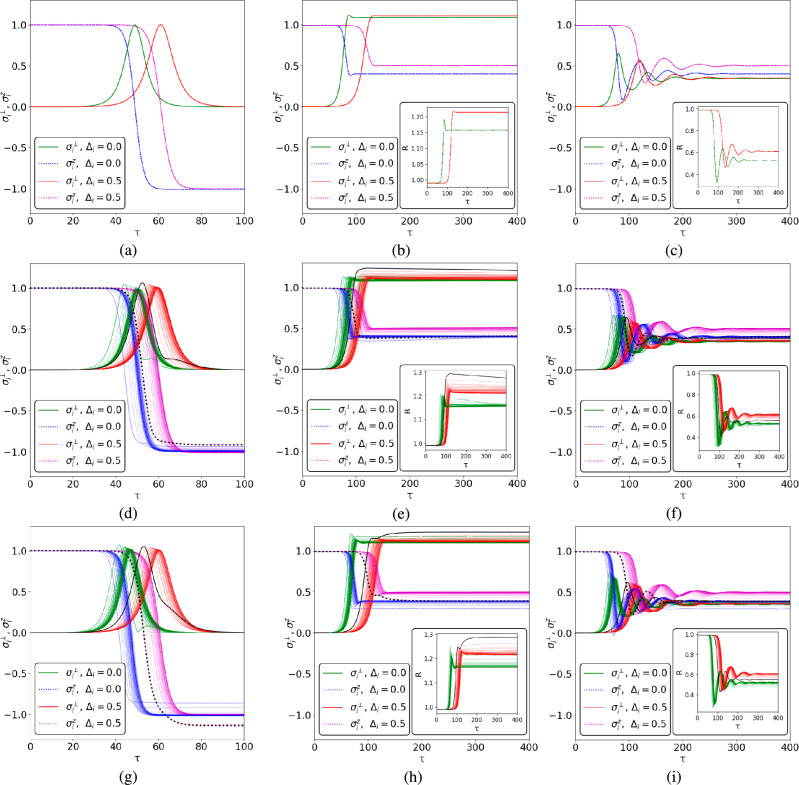
$\sigma ^{\perp }_i$ and $\sigma _i^z$ vs. dimensionless time $\tau = \kappa t$ for a scale-free network with $N = 60$ nodes, see Fig. 2(b). Each curve describes a single node behavior; at (**d**) - (**i**) the black bold curves correspond to the main hub with degree $k_i = 38$ at $\Delta _i = 0.5$. For (**a**) - (**c**) $J = 0$; for (**d**) - (**f**) $J = J_R = 0.01, J_I = 0$; for (**g**) - (**i**) $J = -J_I = -0.01i, J_R = 0$. The parameters are: $\Gamma , \gamma _{+,i}, \sigma _{0,i} = 0$ for (**a**), (**d**), (**g**); $\Gamma = 0.1$; $\gamma _{+,i} = 0.5$; $\sigma _{0,i} = 0.99$ for (**b**), (**e**), (**h**): $\Gamma = 0.1$, $\gamma _{+,i} = 0.05$; $\sigma _{0,i} = 0.99$ for (**c**), (**f**), (**i**). Other parameters are $g/\kappa = 0.5$; $\sigma ^{\perp }_i (\tau = 0) \simeq 0$; $\sigma _i^z(\tau = 0) \simeq 1$.

**Fig. 6:**
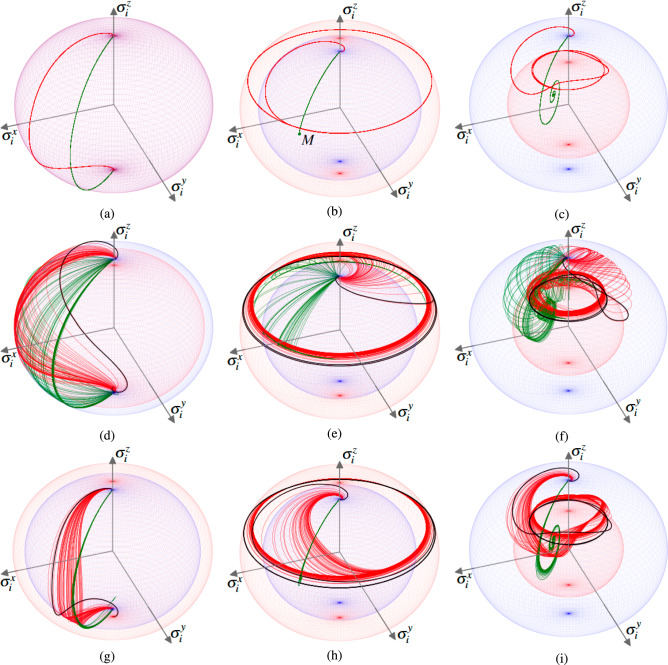
Trajectories of the emotional states on an affective sphere. The thick black curves correspond to the network’s main hub at $\Delta _i = 0.5$. In (**b**) green dot *M* denotes the stationary state. Parameters are the same as in Fig. 5.

Temporal dimensionless dependencies of $\sigma ^{\perp }_i$ and $\sigma ^z_i$ are shown in Fig. 5(a). We assume that initially (at $t = 0$) the *i*-th individual emotional state is maximally activated, $\sigma ^z_i(0) \simeq 0$, and the affective vector is directed toward the *z* axis (aroused state); the affective field is zero in this limit ($\sigma ^{\perp }_i \simeq 0$), see Fig. 6(a). Then, after the individual’s response time $t_d$, the affective vector is projected onto the *XOY* plane. To be more specific, for our simulations, we take $t_d = 0$ in this work. The red curve in Fig. 6(a) performs some rotation of the affective vector in this plane that corresponds to emotional state variation and indicates the existence of a non-zero affective field. Both curves in Fig. 6(a) approach the south pole of the sphere with $\sigma ^z_i = -1$, which indicates a calming (unaroused state) of the individual. Notably, since $R_i = 1$, see (42c), the curves do not leave the affective sphere; the green curve in Fig. 6(a), which is relevant to $\Delta _i = 0$, demonstrates an immediate transition to the south pole of the affective sphere without any rotation.

The situation dramatically changes at non-zero $\gamma _{+,i}$, $\Gamma _{i}$. We represent these results in Fig. 5(b,c) and Fig. 6(b,c).

At $\sigma ^z_{0,i}> \sigma ^z_{cr,i}$ Eqs. (29) admit a limiting cycle solution that we can obtain from the equations below

43a $$\begin{aligned} \dot{\sigma }^x_i&= -\Gamma _i \sigma ^x_i+\sigma ^z_{i}(C_i\sigma ^x_i - D_i\sigma ^y_i); \end{aligned}$$

43b $$\begin{aligned} \dot{\sigma }^y_i&= -\Gamma _i \sigma ^y_i +\sigma ^z_{i}(C_i\sigma ^y_i + D_i\sigma ^x_i); \end{aligned}$$

43c $$\begin{aligned} 0&=(\sigma ^z_{0,i}-\sigma _{i}^z)\gamma _{+,i} - C_i\Big ((\sigma ^x_i)^2 + (\sigma ^y_i)^2\Big ). \end{aligned}$$

We borrow the solution of Eqs. (43a), (43b) in the form

44 $$\begin{aligned} \sigma ^x_i = \sigma ^{\perp }_{0,i} \cos (\omega _{L, i} t); \quad \sigma ^y_i=\sigma ^{\perp }_{0,i} \sin (\omega _{L,i} t) \end{aligned}$$

and substitute it into (43a), (43b) to find the frequency of oscillations as

45 $$\begin{aligned} \omega _{L, i} = \frac{\Gamma _i \Delta _i}{\kappa }. \end{aligned}$$

In Fig. 6(b) and Fig. 6(c) we can see the limiting cycle oscillations with frequency $\omega _{L, i}$, which are accompanied by the affective sphere radius enhancement (Fig. 6(b)) or reduction (Fig. 6(c)). At $\Delta _i>0$ the depicting point on affective sphere experiences a counter-clockwise rotation, as shown in Fig. 6(b) and Fig. 6(c) for the red curves. At $\Delta _i < 0$, a point on affective sphere rotates clockwise around the $\sigma _i^z$ axis. Noteworthy, in the limit $\Delta _i \rightarrow 0$ from (45) we obtain $\omega _{L, i} \rightarrow 0$. In this limit, the representing point on the affective sphere does not experience rotation around the $\sigma _i^z$ axis and occupies some point on the ring; the green curve occupies point *M* in Fig. 6(b) that is placed on some ring (not shown in Fig. 6(b)). Notably, the rings for the red and green curves in Fig. 6(b) possess different radii $\sigma ^{\perp }_i$ due to distinct values of $C_i$, which is detuning dependent, see Eq. (27).

### Collective emotions in the presence of weak AIA – NIA coupling

#### Phase transition for affective social field

Let us now examine the limit of DIS when NIA – AIA pairs, which represent the nodes in Fig. 2, are weakly connected through the complex network. We consider numerical solutions of Eqs. (29) and establish them in Figs. 5(d-i) and Figs. 6(d-i), respectively. To be more specific, for calculations we use the parameters of the graph presented in Figs. 2(b). Thus, each curve in Figs. 5(d-i) and Figs. 6(d-i) relays to some node features in Figs. 2(b); the bold black curves correspond to the users that occupy the largest hub with node degree $k_i = 38$ in Figs. 2(b) at $\Delta _i = 0.5$. To elucidate the network influence on any *i*-th NIA within the DIS, we chose a coupling strength amplitude |*J*| small enough. The behavior of the curves in Fig. 5(d-i) in this limit mimics the basic features of the curves in Fig. 5(a-c), respectively. Here we emphasize some specific features of dependencies in Figs. 5(d-i), 6(d-i), which are specified through the network influence. In particular, for Fig. 5(d-f) and Fig. 6(d-f) we assume that $J_I = 0$. In other words, all NIA – AIA pairs are coupled within the network coherently. In this case, it is easy to see the variety of emotional “trajectories” of individuals on the affective spheres. This variety is clearly seen in Fig. 6(f) as extra (toroidal) oscillations for the red and green curves.

Contrary, Figs. 5(g-i) and Figs. 6(g-i) establish results for active network with $J_R = 0$, $J_I \ne 0$. This limit corresponds to the “pure” diffusion of an individual’s emotion within the network, see Figs. 5(g-i) AIAs. Figs. 6(g-i) clearly demonstrate that external (to network) information reservoir suppresses the emotional variety of NIAs within the network. In particular, at $\Delta _i\ne 0$ the variety of emotional states is reduced, cf. red curves in Figs. 6(d-f) and Figs. 6(g-i), respectively. At the same time, the green lines corresponding to different nodes in Figs. 6(d-f) at $\Delta _i = 0$ are almost collected in a narrow band of trajectories on the affective sphere. We can suppose that such behavior represents the result of emotional state synchronization induced by the network. Let us examine this limit analytically.

Inserting (25) into (26) at $\Delta _i = 0$, $J_R = 0$ we obtain

46 $$\begin{aligned} \dot{p}_i = \mathcal {A}_ip_i - \mathcal {B}_i |p_i|^2 p_i + F_i, \end{aligned}$$

where we introduce

47 $$\begin{aligned} F_i = -\frac{C^2\sigma ^z_{0,i}J_{I,eff}}{g^2} \sum _{j=1}^{N}A_{ij}p_j; \end{aligned}$$

which represents a small force acting on the *i*-th NIA from the other network actors. We can also elucidate the practical meaning of force $F_i$ by the annealed network approach that helps to represent adjacency matrix elements as (cf.^98^

48 $$\begin{aligned} A_{ij} = \frac{k_ik_j}{N\langle k\rangle }. \end{aligned}$$

Substituting (48) into (47) we obtain

49 $$\begin{aligned} F_i = -\frac{C^2\sigma ^z_{0,i}J_{I,eff}}{g^2} k_i\bar{P}. \end{aligned}$$

In (49) we introduce average social field polarization as

50 $$\begin{aligned} \begin{gathered} \bar{P} = \frac{1}{N\langle k\rangle }\sum _{i=1}^{N} k_i p_i. \end{gathered} \end{aligned}$$

Thus, we consider a small perturbation of the emotional state of the *i*-th user from the social field polarization, examining real $p_j$.

Phase $\Theta$ plays a significant role in (47), (49). At $\Theta=0$, the AIA – AIA coupling remains diffusive (lossy). The community of the network with environment extinguishes the emotions of its individuals; external force $F_i$ ($F_i < 0$) helps to achieve NIA’s emotional state, its equilibrium.

Noteworthy, at $\Theta = \pi /2$ the social force $F_i = 0$. Since we suppose above $J_R = 0$, this limit indicates the situation when individuals are not affected by the network as the environment.

At $\Theta = \pi$ ($J_{I,eff} = -J_I$, $F_i> 0$) the network environment becomes active. In this case, external force $F_i$ enhances the moods within the network. In particular, at steady state $\dot{p}_i = 0$, little bit above the threshold point we can assume in Eq. (47) that $p_j\simeq p_i^{(0)}$ is known given by its value (37) and

51 $$\begin{aligned} F_i \equiv F_i^{(0)} \simeq \frac{1}{g^2}C^2\sigma ^z_{0,i}J_I p_i^{(0)} k_i, \end{aligned}$$

where $k_i = \sum _{j}A_{ij}$ is the *i*-th node degree. Thus, at the steady state (46) takes the form

52 $$\begin{aligned} \mathcal {A}_ip_i - \mathcal {B}_i p_i^3 + F_i^{(0)} = 0, \end{aligned}$$

where we introduce vital parameters

53 $$\begin{aligned} \mathcal {A}_i = (C\sigma ^z_{0,i}-\Gamma _i); \quad \mathcal {B}_i = \frac{4C^2}{\gamma _{+,i}}. \end{aligned}$$

Eq. (52) may be recognized in the framework of second-order phase transition for emotional states, cf.^99^. For vanishing external force $F_i^{(0)}\simeq 0$ normal state implies $p_i$ that corresponds to trivial solutions (33). The critical value of arousal we can find from the condition $\mathcal {A}_i=0$; it is described by (35). The affective field parameter $p_i$ may be obtained from (52) as $p_{ i} = \sqrt{\frac{\mathcal {A}_i}{\mathcal {B}_i}}$ and lead immediately to Eq. (37).

Being above very close to the threshold, we can allow $\mathcal {A}_i\simeq 0$ in (52). In this case from (52) we obtain

54 $$\begin{aligned} p_i \simeq \Bigg (\frac{F_i^{(0)}}{\mathcal {B}_i}\Bigg )^{1/3}. \end{aligned}$$

Thus, Eqs. (52)- (54) specify a non-equilibrium second-order phase transition for the affective field $p_i$, recognizing it as an order parameter that specifies individuals emotional level activation (or, deactivation). In particular, we can recognize the normal state as an emotionally neutral one (without arousal component) with $p_i = 0$. The emotional activations create positive arousal with $p_i> 0$.

Eqs. (48)- (50) allow to establish (46) for average social field $\bar{P}$ in the form

55 $$\begin{aligned} \dot{\bar{P}} = \mathcal {A} \bar{P} - \frac{C^2\sigma ^z_{0,i}J_{I,eff}\zeta }{g^2} \bar{P} \end{aligned}$$

where we neglected the difference in $\mathcal {A}_i\simeq \mathcal {A}$ coefficients and also dropped the nonlinear term proportional to the $\mathcal {B}_i$. Solution of (55) is

56 $$\begin{aligned} \bar{P}(t) = e^{\mathcal {A}_{eff}t} \bar{P}(t=0), \end{aligned}$$

where we defined effective (network-enforced) social gain

57 $$\begin{aligned} \mathcal {A}_{eff}=\mathcal {A} - \frac{C^2\sigma ^z_{0,i}J_{I,eff}\zeta }{g^2}=\frac{g^2 \sigma ^z_{0} }{\kappa } \Big (1 - \cos \Theta \frac{J_I\zeta }{\kappa }\Big ) - \Gamma , \end{aligned}$$

where we take $\sigma ^z_{0,i}\simeq \sigma ^z_{0}$, $\Gamma _i\simeq \Gamma$ for all *i*-th NIAs.

Eqs. (56), (57) represent one of the vital results for our work. In particular, (56) establishes the temporal evolution of the average diffusive social field in DIS that determines the collective emotions and moods in the network.

At $\mathcal {A}_{eff} < 0$ these moods are attenuated and tend to their equilibrium. For $\mathcal {A}_{eff}> 0$ it is possible to obtain an enhancement of the moods in the network; in Figs. 5(d-f). It is important that all ingredients of DIS are significant for that. In particular, personal emotional features of NIAs and their coupling with the environment are encoded in $\sigma ^z_{0}$ and $\Gamma$ parameters. The parameter *g* characterizes the effectiveness of NIA – AIA interaction. The $\kappa$ determines AIA’s peculiarities. The network features are specified by the term $J_I\zeta$. The interaction of the network with the environment is determined by $J_I$ and $\Theta$, which promotes enhancement for $\Theta = \pi$. The complex network topology in (57) is determined by the $\zeta$ parameter, that determined by the second-order degree correlation function, see (15). Noteworthy, $\zeta$ can be large enough, $\zeta \gg 1$. In this limit from (57) we obtain $\mathcal {A}_{eff}\propto \zeta$ that indicates opportunity of significant enhancement of collective emotional state and moods in the network within anomalous and scale-free regimes. Such a situation is reminiscent of social laser behavior, cf.^28^.

#### The role of complex network synchronization

As we have seen above, the behavior of the collective green curves in Figs. 6(g-i) allows us to assume that in the vicinity of $\Delta _i \simeq 0$ the synchronization of individual network agents occurs. Let us examine this limit in more detail. We discuss (26) in the limit of negligible nonlinear terms. In this case, the emotional state activation is mainly determined by the coupling with external reservoir, i.e. $\sigma _{i}\simeq \sigma ^z_{0,i}$; from (26) we obtain (cf. (46))

58 $$\begin{aligned} \dot{p}_i = - iD\sigma ^z_{0,i}p_i + \mathcal {A}_ip_i + i\frac{C^2\sigma ^z_{0,i}J}{g^2}\Big (1 - i\frac{D}{C}\Big )^2 \sum _{j=1}^{N}A_{ij}p_j. \end{aligned}$$

Assuming $D/C \ll 1$ and substituting $p_i \simeq re^{\phi _i}$ into (58) we obtain

59 $$\begin{aligned} \dot{\phi }_i = \omega _i + K_i\sum _{j=1}^{N}A_{ij}\sin (\phi _j -\phi _i), \end{aligned}$$

where we introduce the parameters

60 $$\begin{aligned} \omega _i = -D \sigma ^z_{0,i}; \quad K =-\cos \Theta \frac{C^2\sigma ^z_{0,i}J}{g^2}. \end{aligned}$$

Eq. (59) with (60) identifies the familiar Kuramoto model for the complex networks, see e.g.^22,100–102^. Noteworthy, the sign of $\omega _i$ and $K_i$ parameters in (60) can be positive or negative, which depends on the choice of *D* and $\Theta$. For the PLDD networks, this model exhibits a rich behavior depending on the degree exponent $\nu$, see e.g.^100^. It is important that this behavior includes critical features of the network that relate to NIA – AIA pairs synchronization within the complex network of DIS. Namely, the critical coupling for the agents’ synchronization calculated within the annealed network approximation (48) is

61 $$\begin{aligned} K_c = \frac{K_{0}}{\zeta }, \end{aligned}$$

where $K_{0}$ is the Kuramoto’s critical coupling for the onset of synchronization, see e.g.^103^. Eq. (61) manifests vanishing AIA – AIA effective coupling rate *K* with increasing $\zeta$. Noteworthy, we expect that the hubs play a significant role in this dynamics. In particular, emotional states and moods of particular NIAs are synchronized through the AIAs connection in the DIS network.

In this work, we do not consider more rigorous but complicated approaches for complex network synchronization that account for high-order degree correlations, see e.g.^104^. Specific features, stability of such networks over long times, represent a challenging problem for further analysis; we expect that we can perform this analysis in future elaboration.

## Conclusions

Let us summarize the results obtained. In this work, we have adopted a heuristic (quantum physical) approach to describe a distributed intelligent system, based on the Hamiltonian approach for systems of interacting agents within networks. In particular, we have examined the quantum-like model of DIS that represents a two-layer network and consists of *N* pairs of NIAs (users), upper layer in Fig. 3(a), and their assistants (LLM-based agents) AIAs, lower layer in Fig. 3(a). The NIA – AIA interaction is governed by the *g* interaction strength, see Table 1. The AIAs are coupled to each other within the complex (scale-free) network and participate as teammates in DM problems; AIA – AIA dissipative coupling is specified by a complex parameter *J*.

In this work, we examine three major limiting cases for the parameters *g* and *J*. In the first case, with $g = J = 0$, the analysis reduces to the emotional states of individual users, see Quantum-like 3D model of feelings and emotions subsection. We used a 3D PAD model as a baseline to specify the emotional state of individuals. A notable feature of this model is that it can be readily described in the framework of quantum probability theory approaches. We introduce the affective sphere representation for the individuals’ emotional state. An affective sphere admits a simple analogy with the Bloch sphere, which is familiar in quantum theory for describing the features of two-level systems. The main advantage of this description is that it makes it possible to use a well-developed quantum tomography approach as a new way of comprehensively identifying an individual’s quantum-like emotional states.

Second, we examine the limit of $g \ne 0$, $J = 0$, see Network-free individuals emotional states, $J=0$ subsection. We propose modeling AIAs within the DIS using LLM capabilities. Modern LLM-based agents, which are trained using large-scale datasets, can interpret meaning, follow dialogue context, recognize intent, and infer emotional states. However, their outputs are usually informative rather than decisive, as LLM-based agents still lack genuine real-world understanding and the ability to verify facts. LLM-based AIAs predict the next token based on training distributions rather than truth. Consequently, they tend to ’adiabatically’ align with the prevailing discourse and tone of interactions with NIAs, thereby reinforcing their supportive role.

Our approach, which is based on a superradiant quantum-like model, assumes the adiabatic elimination of the information field inherent to the AI system (lower layer in Fig. 3(a)), and AIAs’ individual emotional states are not treated explicitly within this approximation. More precisely, the emotional state of the *i*-th AIA follows their NIAs’ social field, $p_i \simeq \sigma _i^-$, defined in Eq. (26). In this regard, we focus on AIAs-mediated formation and spread of emotions within the NIAs online community that could be described by using a macroscopic average social field that relates to the upper layer in Fig. 3(a).

In the framework of mean-field theory, we examine open (driven-dissipative) DIS as a source of NIAs’ emotional states modification that occurs as a result of their information exchange with AIAs and a huge external information reservoir. In this case, mean-field (classical) trajectories in the affective sphere characterize changes in NIAs’ emotional state. We have demonstrated that, in the absence of interaction with the reservoir, an individual experiencing positive arousal will relax into a calm (unaroused) state over time, Fig. 6(a). In the presence of a permanent influential external reservoir, the emotional states change but do not fully relax. The level of emotional deactivation is determined by a combination of external factors, as well as individual properties of the users. These factors ultimately enable the formation of limiting cycles of emotional behavior, Figs. 6(b,c). More precisely, the combination of these factors meets a specific condition (35) that defines the phase transition of the individual’s emotional state. If this condition is met, the individual’s state is characterized by a certain affective field through which they can influence others.

An important third part of this work is presented in the subsection Collective emotions in the presence of weak AIA – NIA coupling, which examines the limit $g\ne 0$, $J \ne 0$. Assuming weak AIA – AIA coupling, we identify two distinct dynamical regimes in emotional formation across the DIS. In the first regime, coherent AIA – NIA coupling results in diverse emotional state distributions across the network, supporting emotional heterogeneity and individual differentiation. In the second regime, the shared interaction between AIAs and an external information reservoir promotes the coherent synchronization of user emotions. This leads to the emergence of a macroscopic affective field, which encapsulates the collective emotional dynamics within the system.

We draw an analogy between macroscopic quantum phenomena, such as superradiance in quantum physics, and the formation of collective emotions within social networks. In particular, quantum two-level systems exhibit behavior similar to that shown in Figs. 5, 6(a). Ultimately, superradiance (the analogue of the affective field in our work) is achieved in an ensemble of quantum systems by establishing quantum correlations between two-level systems. These correlations are the source of synchronization in the behavior of these systems. The behavior of agents in DIS looks different. Here, the general affective field characterizing collective emotions in the network can be established due to the properties of the network. Under certain conditions, the network helps coherently synchronize individual users. Thus, a complex network’s peculiarities (node degree correlations are coded in the $\zeta$ parameter) practically play the same role for DIS agents as quantum correlations in an ensemble of two-level physical systems. In other words, a quantum-like superradiant state represents a network-induced, emotionally activated collective state emerging within a social community. This conclusion looks intriguing in the study of the behavior of complex social systems. Further analysis of correlation creation in both physical and complex social systems is required; the results of this analysis will be published separately.

## Data Availability

The datasets generated and/or analyzed during the current study are available in the Data-QIMSI repository, https://github.com/DTsarev-ITMO/Data-QIMSI.git
